# Patient desire for spiritual assessment is unmet in urban and rural primary care settings

**DOI:** 10.1186/s12913-021-06300-y

**Published:** 2021-03-31

**Authors:** Joseph R. Fuchs, Jeffrey W. Fuchs, Joshua M. Hauser, Marilyn E. Coors

**Affiliations:** 1 University of Colorado School of Medicine, 13080 E 19th Ave, Office 208B, Aurora, Colorado 80045 USA; 2 Feinberg School of Medicine, Northwestern University, Chicago, IL USA; 3 Division of Palliative Care, Northwestern University Feinberg School of Medicine and Jesse Brown VA Medical Center, Chicago, IL USA; 4 Department of Psychiatry & The Center for Bioethics and Humanities, University of Colorado School of Medicine, Aurora, Colorado USA

**Keywords:** Patient desire, Spiritual assessment, Spirituality, Religion, Urban, Rural

## Abstract

**Background:**

Incorporation of patient religious and spiritual beliefs in medical care has been shown to improve the efficacy of medical interventions and health outcomes. While previous study has highlighted differences in patient desire for spiritual assessment based on patient religiosity, little is known about patient desire for spiritual assessment based on community type, particularly in urban compared to rural communities. We hypothesized that, given demographic trends which show a higher degree of religiosity in rural areas, patients in rural communities will be more likely to desire spiritual assessment.

**Methods:**

In this cross-sectional study of 141 adult primary care patients in rural and urban Colorado at non-religiously affiliated clinics, we surveyed patient demographic information, measures of religiosity, patient desire for spiritual assessment, and frequency of spiritual assessment in practice. Univariate logistic regression analyses were used to compare the two populations.

**Results:**

In both Denver County (urban) and Lincoln County (rural) over 90% of patients identified as religious, spiritual, or a combination of the two. Thirty eight percent (38.3%) of patients in Denver County and 49.1% of patients in Lincoln desired spiritual assessment. Over 97% of patients in both areas reported rarely or never being asked about their R/S within the past year. For patients who have had five or more clinic visits in the past year, more than 91% in both areas stated they have never or rarely been asked about their beliefs.

**Conclusions:**

While the majority of patients in this study identify as religious or spiritual and many patients desire spiritual assessment, the majority of patients have never or rarely been asked about their spirituality within the past year. This demonstrates a significant gap between patient preference and provider practice of spiritual assessment in the primary care setting, which was similar in both rural and urban settings. This highlights the need for interdisciplinary focus on spiritual assessment and incorporation of patient R/S beliefs in medical care to provide holistic patient care and improve health outcomes.

**Supplementary Information:**

The online version contains supplementary material available at 10.1186/s12913-021-06300-y.

## Background

Since the 1990s, the impact of Religion and Spirituality (R/S) on patient health has received increasing attention. This is seen in the exponential growth in publications on the topic of religion, spirituality, and health over the past three decades [1]. Over this time period, more than one thousand studies have examined the impact of R/S on patient health and health outcomes [2, 3]. Though there are mixed data from these investigations, the majority of studies report significant relationships between R/S and improved health [3]. This includes studies which have shown improvement in coping with illness, an inverse link between R/S and depression, and even potential favorable effect on overall survival [3–5]. Further, recent research suggests that the incorporation of patient R/S improves the efficacy of medical interventions and the integration of R/S beliefs in care leads to improved measures of patient hope and quality of life [6, 7].

The beneficial impact of incorporating patient R/S in health care has led to more widespread recognition of the role of spirituality in providing whole person care. This has been codified in various national initiatives, including a requirement by the Joint Commission for spiritual assessment, which has been defined as employing methods to identify a patient’s spiritual suffering and spiritual needs related to medical care [8, 9]. Additionally, the Medical Student Objectives Project of the Association of American Medical Colleges (AAMC) advocates for medical students to develop an understanding of spirituality and learn how to take a spiritual history [10]. Taken together, these guidelines and requirements suggest a goal for the health care team to harness knowledge of patient R/S to improve health outcomes.

Patients’ desire for spiritual assessment has also received significant attention, especially in the context of serious illness and end-of-life care [11–15]. Spirituality in the setting of health care has been defined as the “aspect of humanity that refers to the way individuals seek and express meaning and purpose and the way they experience their connectedness to the moment, to self, to others, to nature, and to the significant or sacred” [16]. Given this definition, it is apparent why such attention has been focused on patients’ desire for spiritual assessment in discussions of serious illness and at end-of-life. In fact, one study found that more than 70% of patients desired a discussion of spirituality in the context of life-threatening illnesses, serious medical conditions, and loss of loved ones [17].

Spiritual assessment, however, is not meant to be used exclusively in serious illness and end-of-life settings. Various studies performed in outpatient and inpatient settings have found that 30–63% of undifferentiated general medicine patients are interested in a discussion of R/S with their provider [17–21]. In order to utilize an understanding of patient R/S to improve health outcomes, the health care team must first obtain this important part of the patient history. Distinct tools have been created for physician and other health care provider use in obtaining a spiritual history [22–24]. There has been some resistance to the implementation of spiritual assessment taking in clinical practice due to concerns about ethical issues such as lack of spirituality training and invasion of privacy [25]. However, the evidence supporting the benefits on patient health outcomes and the recognition of the importance of spiritual assessment by national organizations suggest the need for effective implementation of this practice [17, 25, 26].

While the importance of spiritual assessment is generally recognized, there is a gap in understanding of patient desire for spiritual assessment and its practice in rural and urban settings. Previous research has demonstrated that rural populations have higher levels of religiosity compared to urban populations [27]. Additionally, studies have shown that patients with higher levels of religiosity are more likely to desire spiritual assessment [21]. Therefore, we hypothesize that patients in rural settings will be more likely than patients in urban settings to desire spiritual assessment due to geographic variation in religiosity. We also hypothesize that patients in rural settings will be more likely than their urban peers to have received spiritual assessment, as previous study has shown that patients with higher levels of religiosity are more likely to have discussions about their R/S concerns in health care settings [21].

This study sought to explore patients’ desire for spiritual assessment and its current practice in urban and rural settings.

## Methods

We conducted a survey of patients > 18 years old who receive medical care in non-religiously affiliated primary care offices in Lincoln County, Colorado (population 5610) [28] and Denver County, Colorado (population 716,492) [29] from July to September 2019 and December 2019 to January 2020, respectively. Patients in the waiting rooms of primary care offices were asked to participate in the survey after their medical visit by a study author (JRF) who had no prior relationship with the participants. Every third patient leaving their visit was asked to participate during the study period. This sampling strategy was utilized to avoid potential biases associated with a convenience sampling strategy. Participants were provided a consent form outlining risks, benefits, and overarching goals of the study. The response rate was 78.6% in Denver County and 70.6% in Lincoln County.

The confidential survey was completed by the patients independently in written format. This study was part of a larger survey designed by the authors to understand patients’ experience with and views of spiritual assessment and religiously-affiliated care. Survey questions were created in an iterative process based on previous studies and recommendations of expert colleagues [30, 31]. The preliminary survey draft was beta-tested by 10 non-medical individuals to ensure the questions asked were readable and not ambiguous.

The survey (included as a supplementary file) asked demographic information including age, gender, race/ethnicity, educational attainment, how often the patient visited the clinic over the past year, a binary question about patient identification as religious, and a free response question to specify religious affiliation if applicable. Patients were asked about their level of R/S using two separate Likert scale measures, one for religiosity and one spirituality (“Not at all religious/spiritual” = 0 to “Very religious/spiritual” = 3). Patients were also asked which statement best describes them: “spiritual and not religious,” “religious and not spiritual,” “religious and spiritual,” or “do not believe in either.”

In order to assess patient desire for spiritual assessment, patients were asked “How important is it that the people caring for you in a hospital/clinic/Doctor’s office know about your religious/spiritual beliefs?” with Likert scale measures from “Not important” to “Very important.” Additionally, patients were asked “In the last year how often did anyone in a hospital/clinic/Doctor’s office ask about your religious/spiritual beliefs?” with answer choices “Never,” “Rarely,” “Usually,” or “Always.” In order to assess patient desire for religious concordance with their health care team, patients were asked “Overall how important is it that the people caring for you have the same religious/spiritual beliefs as you?” with Likert scale measures from “Not at all important” to “Very important.”

RStudio 3.6.2 was used to conduct statistical analysis [32]. Descriptive statistics are reported for numeric measures of age, gender, race/ethnicity, educational attainment, religion, and religious affiliation. The descriptive statistics for each location were compared using Fisher’s exact test with significance at *p* ≤ 0.05. Racial/ethnic demographics and religious affiliation categories were consolidated when conducting analyses using the Fisher’s exact test to limit categories with few or zero responses. We utilized univariate logistic regression to compare patient desire for spiritual assessment, religious/spiritual concordance with the health care team, and practice of spiritual assessment between the two locations, which allowed for odds ratios to be determined (*p* ≤ 0.05). This was completed using all individuals as well as for two subsets: (1) only considering patients who identify as spiritual or very spiritual and (2) only considering patients who identify as religious or very religious. Missing data were excluded from analysis and the assumptions for Fisher’s exact test and logistic regression were assessed and met for all analyses.

The survey and methodology were approved with exemption status by the Colorado Multiple Institutional Review Board (COMIRB 19–1704).

## Results

Eighty-one (81) patients in Denver County and 60 patients in Lincoln County completed the survey. Participant characteristics by location are given in Table 1. In Denver County, 69.1% of patients surveyed were female, 86.3% identified as White/Caucasian (Non-Hispanic), and the average age at the time of data analysis was 57 years (± 17.8 years). In Lincoln County, 68.3% of patients surveyed were female, 93.3% identified as White/Caucasian (Non-Hispanic), and the average age was 49 years (± 19.0 years). Educational attainment was significantly different between locations (*p* < .001) with 33.3% of patients in Lincoln County having a high school diploma or GED and 6.7% have attended or graduated from graduate school. In Denver County, 6.2% of patients had a high school diploma or GED while 35.8% attended or graduated from graduate school. Age difference was also statistically significant (*p* = 0.02), with 30.0% of patients in Denver County born in 1950 or before and 11.9% of patients in Lincoln County born in 1950 or before.

**Table 1:** Patient Demographics

	Rural: Lincoln County No. (%)	Urban: Denver County No. (%)	Fisher’s Exact Test ***P***-value**
**Gender**	*n* = 60	*n* = 81	
**Male**	19 (31.7)	25 (30.9)	*P*-value 1.0
**Female**	41 (68.3)	56 (69.1)	
**Year Born**	*n* = 59	*n* = 80	
**1991–2002**	9 (15.3)	7 (8.8)	***P-value 0.02***
**1971–1990**	22 (37.3)	17 (21.3)	
**1951–1970**	21 (35.6)	32 (40.0)	
**1950 or Before**	7 (11.9)	24 (30.0)	
**Race/Ethnicity**^**a**^	*n* = 60	*n* = 80	
**White**	56 (93.3)	69 (86.3)	*P*-value 0.27
**Other**	4 (6.7)	11 (13.7)	
Hispanic	1 (1.7)	3 (3.8)	
Asian/PI	0 (0)	0 (0)	
Black	0 (0)	4 (5.0)	
American Indian	1 (1.7)	2 (2.5)	
Pacific Islander	0 (0)	0 (0)	
Other Specified	0 (0)	1 (1.3)	
Multiple	2 (3.3)	1 (1.3)	
**Educational Attainment**	*n* = 60	*n* = 81	
**Some High School/GED**	20 (33.3)	5 (6.2)	***P-value < 0.001***
**Some College/College**	36 (60.0)	47 (58.0)	
**Some Graduate/Graduate**	4 (6.7)	29 (35.8)	
**Identify as Religious**	*n* = 60	*n* = 81	
**Yes**	43 (71.7)	60 (74.1)	*P*-value 0.85
**No**	17 (28.3)	21 (25.9)	
**Specified Religion**	*n* = 58	*n* = 80	
**Non-denominational** **Christian**	18 (31.0)	17 (21.3)	*P*-value 0.21
**Protestant**	14 (24.1)	14 (17.5)	
Episcopalian	0 (0)	2 (2.5)	
Baptist	2 (3.4)	3 (3.8)	
Presbyterian	0 (0)	2 (2.5)	
Lutheran	3 (5.2)	3 (3.8)	
Methodist	9 (15.6)	4 (5.0)	
**Catholic**	7 (12.1)	15 (18.7)	
**Other**	3 (5.2)	12 (15.0)	
Buddhist	0 (0)	1 (1.2)	
Latter Day Saint	1 (1.7)	0 (0)	
Jewish	1 (1.7)	5 (6.3)	
Specified Other	1 (1.7)	6 (7.5)	
**None**	16 (27.6)	22 (27.5)	

^**a**^Bolded categories were utilized for Fisher’s exact test

**Statistically Significant *P*-values in bold and italicized

Compared to the general population of each county based on US Census Data, patients sampled across both locations were more likely to be female (Denver County: 69.1% study vs. 49.8% US Census, Lincoln County: 68.3% study vs. 41.6% US Census) and more likely to be White (Denver County: 86.3% study vs. 80.8% US Census and Lincoln County: 93.3% study vs. 89.7% US Census) [28, 29]. The majority of patients in each location identified with a Christian denomination (57.5% in Denver County and 67.2% in Lincoln County) which is consistent with the percentage of individuals identifying as Christian in Colorado as described by the 2014 Religious Landscape Survey (64% ± 5.5) [33].

In terms of religiosity and religious affiliation, in Lincoln County, 71.7% of patients identified as religious with 67.2% of patients identifying with a Christian denomination. One patient identified as Jewish and one patient identified as Latter Day Saint. In Denver County, 74.1% of patients identify as religious with 57.5% identifying with a Christian denomination. In Denver County, five patients identified as Jewish, one identified as Buddhist, and three endorsed affiliation with multiple religious denominations. As seen in Table 2, between the two locations, measures of religiosity were significantly different (*p* = 0.03), with 78.2% of patients in Lincoln County and 53.8% of patients in Denver County regarding religion as important or very important in their life.

**Table 2:** Measures of patient religiosity/spirituality and clinical preferences regarding faith

	Rural: Lincoln County No. (%)	Urban: Denver County No. (%)	Fisher’s exact test ***P***-value* (if applicable)
**Importance of Religion**	*n* = 55	*n* = 80	
**Not at all**	5 (9.1)	14 (17.5)	***P-value 0.03***
**Not too important**	7 (12.7)	23 (28.8)	
**Important**	22 (40.0)	19 (23.8)	
**Very Important**	21 (38.2)	24 (30.0)	
**Spirituality Rating**	*n* = 55	*n* = 81	
**Not at all spiritual**	4 (7.3)	7 (8.6)	*P*-value 0.65
**Not too spiritual**	8 (14.5)	17 (21.0)	
**Spiritual**	31 (56.4)	37 (45.7)	
**Very Spiritual**	12 (21.8)	20 (24.7)	
**Best Statement Regarding** **One’s Spirituality and Religion**	*n* = 55	*n* = 81	
**Spiritual and not religious**	15 (27.3)	39 (48.1)	*P*-value 0.10
**Religious and not spiritual**	3 (5.5)	4 (4.9)	
**Religious and spiritual**	33 (60.0)	34 (42.0)	
**Don’t believe in either**	4 (7.3)	4 (4.9)	
**Clinic Visits in the Past Year**	*n* = 55	*n* = 81	
**1 or less**	11 (20.0)	6 (10.9)	*P*-value 0.14
**2–4**	21 (38.2)	31 (56.4)	
**5–9**	14 (25.4)	23 (41.8)	
**> 10**	9 (16.4)	21 (38.2)	
**Occasions in the Past Year Asked** **About Religion and/or Spirituality**	*n* = 52	*n* = 80	
Never	43 (82.7)	63 (78.8)	
Rarely	8 (15.4)	15 (18.8)	
Usually	1 (1.9)	1 (1.2)	
Always	0 (0.0)	1 (1.2)	
**Importance of Provider Knowing** **About One’s Beliefs**	*n* = 55	*n* = 81	
Not important	28 (51.0)	50 (61.7)	
A little important	18 (32.8)	15 (18.5)	
Important	5 (9.1)	13 (16.0)	
Very Important	4 (7.3)	3 (3.7)	
**Importance of Provider Sharing** **the Same Beliefs**	*n* = 55	*n* = 80	
Not at all important	42 (76.4)	68 (85.0)	
Somewhat important	8 (14.5)	7 (8.8)	
Important	4 (7.3)	4 (5.0)	
Very Important	1 (1.8)	1 (1.2)	

*Statistically significant *P*-values in bold and italicized

Ninety-eight percent (98.1%) of patients in the Lincoln County and 97.6% of patients in Denver County had never or rarely been asked about their religion or spirituality in the past year (Table 3). For patients who had 5 or more visits within the past year, 91.3% of patients in Lincoln County and 95.5% of patients in Denver County had never or rarely been asked.

**Table 3:** Desire for spiritual assessment, desire for religious/spiritual concordance, and spiritual assessment in practice for all patients with regression models

	Rural No. (%)	Urban No. (%)	OR (95% CI) (Urban/Rural)
**All Patients**			
**Importance of health care team knowing about one’s beliefs is at least somewhat important**	27 (49.1%)	31 (38.2%)	0.8 (0.5–1.4)
**Importance of having the same belief as one’s health care team is not at all important**	42 (76.4%)	68 (85.0%)	**4.0 (1.6–10.7)**
**Patients never or rarely asked about Religion/Spirituality in clinic in the past year**	51 (98.1%)	78 (97.6%)	0.5 (0.02–4.1)

The majority of patients in Lincoln County (76.4%) and Denver County (85.0%) stated that it was not at all important for their provider to share their same beliefs. This difference was statistically significant, with patients in Denver County more likely to state that having a provider who shared their same beliefs was not important compared to patients in Lincoln County (OR 4.0 (1.6–10.7))**.** Of patients who stated it was at least somewhat important for their provider to know about their faith, 25.8% of patients in Denver County and 37.0% in Lincoln County also said they preferred a provider with the same beliefs. When comparing these patient groups in Denver County to those in Lincoln County, there was no significant difference (OR 0.37 (0.1–1.1)).

Forty-nine percent (49.1%) of patients in Lincoln County and 38.2% of patients in Denver County thought it was at least somewhat important for their health care team to know about their beliefs. There was no significant difference between the geographic locations for patient desire for spiritual assessment (OR 0.8 (0.5–1.4)). When limited to those who call themselves “Spiritual” or “Very Spiritual,” 52.4% of patients in Lincoln County and 50.9% in Denver County desired spiritual assessment (Table 4). For patients who identify as “Religious” or “Very Religious,” 55.8% in Lincoln County and 53.5% in Denver County desires spiritual assessment.

**Table 4:** Desire for spiritual assessment and desire for religious/spiritual concordance of spiritual or religious patients with regression models

	Rural No. (%)	Urban No. (%)	OR (95% CI) (Urban/Rural)
**Spiritual or Very Spiritual**			
**Importance of health care team knowing about one’s beliefs is at least somewhat important**	22 (52.4%)	29 (50.9%)	2.0 (0.9–4.5)
**Importance of having the same belief as one’s health care team is not at all important**	30 (71.4%)	45 (73.7%)	0.8 (0.3–2.0)
**Religious or Very Religious**			
**Importance of health care team knowing about one’s beliefs is at least somewhat important**	24 (55.8%)	23 (53.5%)	1.3 (0.6–3.1)
**Importance of having the same belief as one’s health care team is not at all important**	30 (69.8%)	32 (76.2%)	0.8 (0.3–2.0)

## Discussion

The results of the study indicate that approximately 40% of patients in the outpatient general medicine settings in both an urban (Denver County) and rural (Lincoln County) location desire spiritual assessment. This is consistent with studies conducted over the past 20 years which have found that between 30 and 63% of outpatients desire for providers to know about their beliefs [17–19]. This suggests that although religious demographics of the US population have changed over time, patient desire for spiritual assessment has remained relatively stable [34].

We hypothesized that patients in the rural location (Lincoln County) would be more likely than their urban peers to desire spiritual assessment due to geographic differences in religiosity. Patients in rural Lincoln County had significantly higher ratings of religiosity compared to the patients in urban Denver County. We found, however, that patient desire for spiritual assessment was not significantly different between Denver County and Lincoln County. This suggests that patient desire for spiritual assessment in the primary care setting may not vary by geographic or community setting.

While patients in Lincoln County found religion to be significantly more important than patients in Denver County, in both counties more than 90% of patients identified as religious, spiritual, or a combination of the two. In addition, approximately 50% of patients identifying as either religious or spiritual desired their provider to know about their R/S beliefs. These data suggest the overwhelming majority of patients find R/S to play a role in their lives and patients who identify as spiritual are similarly as likely as their religious peers to desire spiritual assessment. Over the past decade, the number of individuals in the United States who identify as spiritual but not religious has increased by almost 10% [35]. The increasing population of individuals who identify as spiritual but not religious and their desire for spiritual assessment should be considered in health care. As demonstrated in the results of this study, a lack of a religious affiliation does not preclude desire for spiritual assessment.

Although the majority of patients in this study identified as spiritual or religious and many patients found it important for their provider to know about their beliefs, over 97% of patients in both Lincoln County and Denver County reported not being asked about their R/S within the past year. This is true even for patients who have had five or more clinic visits in the past year with 91.3% and 95.5% of patients in Lincoln County and Denver County, respectively, stating that they never or rarely have been asked about their beliefs. This disproves our hypothesis that patients in the rural setting would be more likely to have received spiritual assessment in their health care. It is notable that even though patients in Lincoln County were more religious than patients in Denver County and previous investigations have shown that patients in rural areas are more likely to find religion important [27, 36], there was no difference between provider practice of spiritual assessment between the urban and rural settings. This gap between patient desire for spiritual assessment and the actual practice of spiritual assessment in both the rural and urban settings is concerning. While this result could possibly be explained by utilization of existing knowledge of patients’ R/S beliefs, the focus of spiritual assessment is on incorporating spiritual needs into medical care. Adequate spiritual assessment necessitates inquiring about patients’ beliefs and how they impact patients’ health over time.

Despite accreditation bodies such as the Joint Commission requiring a spiritual assessment and AAMC guidance on spiritual history taking skills for medical students [8, 10], patients in this study reported that spiritual assessment is not regularly conducted by their health care team. Spiritual history taking, one type of spiritual assessment, has been created for physicians and other clinicians to employ in order to assess patient R/S [22–24]. Spiritual history taking can help the provider build rapport with patients, further develop the patient-provider relationship, and elicit important information in the model of whole person care to improve health outcomes. However, a previous study of providers in the Adventist Health System found that while 45–55% of providers find spiritual history taking important, only 11–17% regularly take a spiritual history [31]. The recognition of the importance of spiritual assessment is clear by both providers and patients but is nonetheless infrequently performed. Therefore, additional efforts should be made to ensure that patient R/S is appropriately integrated into care. Notably, spiritual assessment can be conducted by many members of the health care team, including but not limited to nurses, chaplains, social workers, advanced practice providers, and physicians. Forms of spiritual assessment can include brief assessments like the FICA and HOPE models, R/S lifemaps, and comprehensive spiritual assessment [37]. All members of the health care team should be aware of the importance of R/S to patients’ health and be educated in appropriate methods of spiritual assessment.

More than 70% of patients in both Lincoln County and Denver County stated that it was not at all important if their provider held their same R/S beliefs, with patients in Denver County more likely to state that this was *not* important (OR 4.0 (1.6–10.7)). This may be due to greater religiosity ratings in Lincoln County and previous study demonstrating a desire for mutual physician-patient understanding of R/S [17]. Of patients who said it was at least somewhat important for their provider to know about the beliefs, only 37.0% of patients in Lincoln County and 25.8% of patients in Denver County preferred a provider with the same beliefs. Our data demonstrate that a large majority of patients feel it is important for providers to know about their R/S beliefs no matter the provider’s R/S beliefs. This is an important distinction. Patients desire spiritual assessment regardless of religious or spiritual concordance with their health care team.

### Limitations

There are limitations to this study. Overall, the patients sampled across both locations were more female and white than the respective populations in each county based on US Census Data. This limits the interpretation of the results as applied to the counties studied. However, the results can be interpreted in the setting of each individual clinic. In future study, a larger sample size drawn from additional clinics would aid in the interpretation of the results in the context of the counties overall.

Social desirability bias may have affected the results of this study with individuals rating importance of spirituality or religion more highly due to perceived social norms. However, these surveys were anonymous and completed individually by respondents making it unlikely that measures of spirituality or religiosity were overrated. Additionally, recall bias may have caused more patients to believe they have not received spiritual assessment. We chose to ask patients whether they received spiritual assessment in the past year to limit the potential for this bias. Notably, more than 90% of patients who had five or more health care visits within the past year had rarely or never had been asked about their faith or beliefs. Therefore, it is unlikely that the result indicating spiritual assessment was rarely conducted is simply because patients had few health care visits.

This study was conducted at non-religiously affiliated health care facilities. This could underestimate the percentage of patients who desire spiritual assessment for both locations. However, the study sites were chosen to avoid overestimating desire for spiritual assessment by sampling patients who may seek religiously affiliated health care. Finally, the maximum number of patients identifying with a religion other than Christianity in both settings was approximately 15%. There may be differences in patient desire for spiritual assessment based on specific religious affiliation which this study did not address. Rural and urban settings with greater populations of non-Christian identifying patients should be studied to better characterize desire for spiritual assessment.

### Further study

This study explores patient perspectives of spiritual assessment in a sample of patients in urban and rural Colorado. The patients surveyed in this study were majority white, identified as Christian, and were older than 50 years. Further investigation should include broader diversity of patient demographics and R/S beliefs. A national sample would not only improve understanding of patient preferences but could also allow for generalizability of results.

This study demonstrates a desire for spiritual assessment by patients in urban and rural settings and identifies a gap between patient desire for spiritual assessment and its practice. The results of this study cannot fully elucidate the reason for these patient preferences and provider practices. Qualitative studies exploring patient desire for spiritual assessment have found that patients want a holistic approach to medical care and feel that spirituality is connected to healing [38]. However, many of these studies were conducted more than a decade ago and studied religiously affiliated populations [17, 39, 40]. In this work we found that approximately 50% of patients who identify as spiritual desire spiritual assessment. As the proportion of the United States population who identify as “spiritual but not religious” increases, additional qualitative evaluation is needed in order to fully characterize the reasons behind patient desire for spiritual assessment. This may be best accomplished using semi-structured interviews and focus groups.

## Conclusion

Approximately 40% of patients in the outpatient general medicine setting found it important for their provider to know about their R/S beliefs. This desire for spiritual assessment was not dependent on urban or rural location of the clinic. Although many patients desire spiritual assessment, over 97% of patients in both clinic settings have rarely or never been asked about their R/S beliefs within the past year. Given the number of patients who identify as R/S and percentage of patients who desire for their provider to know about their beliefs, we propose that spiritual assessment be performed as a regular component of care in the outpatient general medicine setting. The health care team should utilize spiritual assessment to build rapport with patients, gain valuable information about patient beliefs in the model of whole person care, and harness this knowledge of patient R/S to improve health outcomes.

## Supplementary Information

**Additional file 1.**

## Data Availability

The datasets used and/or analyzed during this study are available from the corresponding author on reasonable request.

## Abbreviations

R/S: Religion and Spirituality
AAMC: Association of American Medical Colleges
