# Supplementary material for: Patient desire for spiritual assessment is unmet in urban and rural primary care settings
Source: BMC Health Serv Res. 2021 Mar 31;21:289. doi: 10.1186/s12913-021-06300-y (PMC8011106; doi:10.1186/s12913-021-06300-y)
Supplement: Supplementary file 1 — Additional file 1. [file 12913_2021_6300_MOESM1_ESM.docx]

In what year were you born? _______________

Do you consider yourself… (pick one)

Male

Female

Other. Please specify_________________________________

Which race/ethnicity best describes you? (Select one

Hispanic

White/Caucasian (non-Hispanic)

Asian/Pacific Islander

Black/African-American (non-Hispanic)

American Indian or Alaskan Native

Pacific Islander

Other single ethnicity. Please specify ___________________________________

Multiple ethnicity. Please specify. ______________________________________

What is the highest level of formal education you have completed?

9^th^ grade to 12^th^ grade

High school diploma or GED

Some college

Graduated college

Some graduate school

Completed graduate degree

What is the zip code of the place where you currently live? ________________

NA, I am currently homeless

Do you identify with a religion?

Yes

No

If yes, in the space below please indicate your religion:

________________________________________________________________

In an average year how often do you attend religious services not including weddings and funerals?

Zero times

Once or twice a year

Every month

Every week or more

Overall, how important is religion in your life?

Not at all important

Not too important

Important

Very Important

Which of the following best describes your level of spirituality?

Not at all spiritual

Not too spiritual

Spiritual

Very spiritual

Which of following statements best describes you? (select one)

I am spiritual but not religious

I am religious but not spiritual

I am both religious and spiritual

I don’t believe in religion or spirituality

In the last year how many times have you gone to a hospital/clinic/Doctor’s office to get care for a health-related issue? ________ # of times.

None- Please skip next question

In the last year how often did anyone in a hospital/clinic/Doctor’s office ask about your religious/spiritual beliefs?

Never

Rarely

Usually

Always

How important is it that the people caring for you in a hospital/clinical/Doctor’s office know about your religious/spiritual beliefs?

Not important

A little important

Important

Very important

Overall how important is it that the people caring for you have the same religious/spiritual beliefs as you?

Not at all important

Somewhat important

Important

Very important
